# Early surgical practice in obstetrics and gynecology specialization: Insights from the first 18 months

**DOI:** 10.1097/MD.0000000000043241

**Published:** 2025-07-04

**Authors:** Sercan Kantarci, Hüsnü Onur Durmaz, Alaattin Karabulut

**Affiliations:** a Department of Obstetrics and Gynecology, Tepecik Training and Research Hospital, Izmir, Turkey; b Department of Obstetrics and Gynecology. Private Medinova Hospital Aydin, Turkey.

**Keywords:** hysterectomy, minimally invasive gynecological surgery, surgical learning curve

## Abstract

This study aims to assess the surgical practices, learning curve, and outcomes of 2 obstetrics and gynecology specialists during the first 18 months of their careers, focusing specifically on minimally invasive gynecological procedures. Data from 277 procedures performed by 2 recently specialized gynecologists in Turkey during the first 18 months of their specialization were retrospectively analyzed. Procedures included total laparoscopic hysterectomy (TLH), vaginal hysterectomy (VH), abdominal hysterectomy (TAH), and other gynecological surgeries. Surgical cases were divided into three 6-month periods, and outcomes such as surgical indications, operative time, length of hospital stay, and complications were evaluated. The study included 277 procedures performed during the first 18 months of a newly specialized obstetrics and gynecology specialist’s practice. TLH was the most frequently performed procedure, with 140 cases, followed by VH with 35 cases and total abdominal hysterectomy (TAH) with 22 cases. The study showed a significant reduction in major complications, with only 4 recorded over the 18 months, including bowel injury and bladder injury. Minor complications, such as wound infections and hematuria, were reported in 8 cases (2.9%), and these decreased significantly over time (*P* = .01). Operation times were shortest in the TLH group (99.6 minutes), and the average hospital stay was shortest for TLH (1.6 days). Overall, 98.6% of surgeries were completed without major complications, with a 100% success rate in the last 6 months. This study highlights the challenges and successes of early surgical practice in gynecology. TLH emerged as the preferred method due to its advantages, while VH was emphasized as the preferred approach when feasible. Regular simulation training, higher case volumes during residency, and mentorship contributed to successful outcomes. These findings underscore the importance of structured training and continuous practice in advancing competency in minimally invasive gynecological surgery.

## 1. Introduction

Gynecological surgery has undergone a significant transformation in recent decades, with the advent of minimally invasive techniques, such as laparoscopy and vaginal surgery. These minimally invasive approaches offer numerous advantages over traditional open surgery, including reduced postoperative pain, shorter hospital stay, and improved cosmetic outcomes.^[1]^ As a result, laparoscopy has become increasingly prevalent in the management of a wide range of gynecological conditions.

The learning curve for laparoscopic surgery can be steep and requires dedicated training and practice to achieve proficiency. However, the benefits of laparoscopy outweigh its initial challenges. With experience and mentorship, surgeons can develop the skills necessary to safely and effectively perform complex laparoscopic procedures.^[2]^

Common complications of hysterectomy include urinary tract damage, bleeding, vaginal cuff dehiscence, wound infection, and gastrointestinal tract injuries.^[3]^ Although laparoscopic hysterectomy has many advantages, vaginal hysterectomy (VH) should be the first choice if it can be performed in appropriate indications.^[4]^

This article examines the challenges, solution strategies, and learning curve encountered during the first 18 months of an obstetrics and gynecology specialist’s career, particularly in the context of benign gynecological surgeries. The focus is on the unique difficulties faced while performing various surgical procedures and the methods employed to overcome these challenges. The development of surgical skills, the impact of resource limitations, and the strategies used to address these obstacles are explored, with a particular emphasis on minimally invasive techniques. Additionally, the role of benign gynecological surgeries in the professional growth of obstetricians and gynecologists is analyzed, along with a broader discussion on the significance of minimally invasive surgery in gynecological care.

## 2. Materials and methods

This retrospective study systematically examines the initial experiences and challenges encountered in performing benign gynecological surgeries during the first 18 months of professional practice as a specialist in gynecology and obstetrics. All surgeries were performed by 2 surgeons with comparable levels of expertise, both in their first 18 months of specialization. Cases with malignancy in the final pathology results, emergency gynecological surgeries, and procedures performed due to obstetric hemorrhage were excluded. During this period, a structured approach was followed in selecting the appropriate hysterectomy method. Vaginal hysterectomy was prioritized whenever feasible, while laparoscopic hysterectomy was chosen as the second option when vaginal surgery was not suitable. Abdominal hysterectomy was performed in patients who could not receive general anesthesia or had a uterine size exceeding 20 weeks of gestation. The selection of vaginal Natural Orifice Transluminal Endoscopic Surgery (V-NOTES) hysterectomy cases was based on specific criteria, including the presence of Grade 1 apical prolapse and the absence of more than 1 previous surgical history.

During the first 18 months of their specialization, 2 gynecologists and obstetricians performed a total of 140 total laparoscopic hysterectomy (TLH), 35 vaginal hysterectomies, 22 abdominal hysterectomies, 4 conversions from laparoscopy to laparotomy (LS > LT), 22 transobturatory tapes, 10 lateral suspensions, and 4 V-NOTES. Data from 2 L/S Burch Colposuspension and 38 laparoscopic bilateral tubal ligation (BTL) cases were recorded. The demographic data of the patients, including age, birth history, surgery history, additional diseases, and body mass index, were recorded.

The first 10 TLH cases were performed collaboratively by 2 obstetrics and gynecology specialists. Subsequent cases were conducted by a single obstetrics and gynecology specialist, with the assistance of a surgical nurse. The cases were categorized into 3 groups: the first 6 months, 6 to 12 months, and 12 to 18 months. Surgical indications, length of hospital stay, blood loss, major complications (including ureteral injury, bladder injury, gastrointestinal system damage, and complications requiring re-surgery), and minor complications (such as hematuria, wound infection, and vaginal cuff dehiscence) were assessed across these 3 time periods.

The data in this article were derived from cases performed by 2 obstetrics and gynecology specialists during the first 18 months of their careers, at a hospital specializing in obstetrics and gynecology with over 3500 births annually and a high volume of gynecological cases, conducted by specialists with similar experience.

Ethics committee approval was received (approval number 2024/07-12) all variables were compared between these groups, and the effect of experience on the surgical outcomes was evaluated. Statistical analysis: Means are expressed as mean (±SD). The number of subjects and rates are given as n (%). Descriptive statistics were applied to all the data. All variables between the groups were evaluated for normality and homogeneity. The Kruskal–Wallis test was performed for non-parametric continuous variables, and Anova test was performed for parametric continuous variables. Statistical significance was set at *P* < .05. In the post hoc analysis, the Bonferroni test was performed for variables that were homogeneously distributed, and the Tamhane test was performed for variables that were not homogeneously distributed. The chi-squared test was used for categorical variables. Statistical analysis was performed using SPSS (version 22.0; Chicago, III, USA).

## 3. Result

The mean age of patients was 54.7 (±9.6) years in the TLH group, 58.1 (±8.6) years in the TAH group, and 60 (±8.9) years in the VH group (*P* = .07). BMI values were 28.7 (±7.8) kg/m² in the TLH group, 27.1 (±9.5) kg/m² in the TAH group, and 29.1 (±7.7) kg/m² in the VH group, with no significant difference among the groups (*P* = .06). The mean gravida was significantly different among the groups, at 2.56 (±0.7) in the TLH group, 3.9 (±0.9) in the TAH group, and 4.9 (±1.6) in the VH group (*P* < .05). Hospital stay duration differed significantly among the groups (*P* < .05), with the TLH group averaging 1.6 (±0.4) days, the TAH group 2.2 (±1.9) days, and the VH group 2.4 (±1.5) days. Hemoglobin decrease also differed significantly (*P* < .05), averaging 1.5 (±0.6) g/dL in the TLH group, 1.9 (±0.6) g/dL in the TAH group, and 1.6 (±0.7) g/dL in the VH group. Operation time was significantly shorter in the TLH group (99.6 (±31.5) minutes) compared to the TAH (130.6 (±19.6) minutes) and VH (136.0 (±22.7) minutes) groups (Table 1**).**

**Table 1:** Demographic and clinical features.

	TLH n = 140	TAH = 35	VH = 22	*P*-value
Age	54.7 (±9.6)	58.1 (±8.6)	60 (±8.9)	*P* = .07
BMI, kg/m^2^ ± (SD)	28.7 (±7.8)	27.1 (±9.5)	29.1 (±7.7)	*P* = .06
Gravide	2.56 (±0.7)	3.9 (±0.9)	4.9 (±1.6)	*P* < .05
Stay hospital, d ± (SD)	1.6 (±0.4)	2.2 (±1.9)	2.4 (±1.5)	*P* < .05
Decrease in hemoglobin, g/dL ± (SD)	1.5 (±0.6)	1.9 (±0.6)	1.6 (±0.7)	*P* < .05
Operation time, min ± (SD)	99.6 (±31.5)	130.6 (±19.6)	136.0 (±22.7)	*P* < .05

SD = standard deviation, TAH = total abdominal hysterectomy, TLH = total laparoscopic hysterectomy, VH = vaginal hysterectomy.

During the first 18 months of surgical practice in obstetrics and gynecology, a total of 277 procedures were documented. These procedures were categorized by type and 6-month periods in which they were performed. TLH was the most frequently performed procedure, accounting for 140 cases. In the first 6 months, 38 TLH cases were performed, followed by 45 in the second 6 months, and 57 in the third 6 months. Vaginal Hysterectomy (VH) was performed a total of 35 times, with 13 cases in the first 6 months, 15 in the second 6 months, and 7 in the third 6 months. Total abdominal hysterectomy (TAH) was performed in 22 cases, with 9 cases in the first 6 months, 7 in the second 6 months, and 6 in the third 6 months.

Conversion from laparoscopy to laparotomy (LS > LT) occurred in 4 cases: 2 cases in the first 6 months, 1 case in the second 6 months, and 1 case in the third 6 months. A total of 22 Transobturator Tape (TOT) procedures were performed: 12 in the first 6 months, 7 in the second 6 months, and 3 in the third 6 months. Lateral Suspension procedures were performed 10 times, including 1 case in the first 6 months, 3 in the second 6 months, and 6 in the third 6 months. BTL was conducted 38 times, with 14 cases in the first 6 months, 13 in the second 6 months, and 11 in the third 6 months. V-NOTES procedures were performed 4 times, with no cases in the first 6 months, 1 case in the second 6 months, and 3 cases in the third 6 months. Lastly, 2 L/S Burch Colposuspension procedures were conducted, with 0 cases in the first 6 months, 1 case in the second 6 months, and 1 case in the third 6 months. These data highlight the distribution and frequency of various surgical procedures over time, showing an increase in the number of TLH cases while other procedures remained relatively stable (Table 2**).**

**Table 2:** Distribution of surgical diversity by month.

	First 6 mo	6–12 mo	12–18 mo	Total
TLH	38	45	57	140
VH	13	15	7	35
TAH	9	7	6	22
Conversion	2	1	1	4
TOT	12	7	3	22
L. Suspension	1	3	6	10
BTL	14	13	11	38
VNOTES	0	1	3	4
Burch	0	1	1	2
Total	89	93	95	277

BTL = bilateral tubal ligation, L. suspension = lateral suspension, TAH = total abdominal hysterectomy, TLH = total laparoscopic hysterectomy, TOT = transobturator tape, V-NOTES = vaginal natural orifice transluminal endoscopic surgery, VH = vaginal hysterectomy.

During the first 18 months of surgical practice, 277 procedures were analyzed, and 98.6% were completed without major complications (Table 3**).** In the first 6 months, 89 surgeries were performed, with a 97.8% success rate. In the second 6 months, 93 surgeries were completed with a 97.8% success rate. In the third 6 months, all 95 surgeries were performed without complications, achieving a 100% success rate. A total of 4 major complications were recorded. In the first 6 months, 1 bowel injury and 1 re-laparotomy were reported. In the second 6 months, 2 bladder injuries occurred. No major complications were observed in the third 6 months (*P* = .07**)** (Table 4). Minor complications were recorded in 8 cases (2.9%), including 3 wound infections (1.1%), 5 hematuria cases (1.8%), and 2 vaginal cuff hematomas (0.7%), mostly occurring during the first 6 months. Minor complications showed a significant decrease over time (*P* = .01) (Table 5).

**Table 3:** Relationship between major complication and type of surgery.

	No complication	Bladder injury	Ureter Injury	Re-laparotomy	Intestinal injury	Total
TLH	138	0	0	1	1	140
VH	34	1	0	0	0	35
TAH	22	0	0	0	0	22
Conversion	4	0	0	0	0	4
TOT	22	0	0	0	0	22
L. suspension	10	0	0	0	0	10
BTL	38	0	0	0	0	38
VNOTES	4	0	0	0	0	4
Burch	1	1	0	0	0	2
Total	273	2	0	1	1	277

BTL = bilateral tubal ligation, L. suspension = lateral suspension, TAH = total abdominal hysterectomy, TLH = total laparoscopic hysterectomy, TOT = transobturator tape, V-NOTES = vaginal natural orifice transluminal endoscopic surgery, VH = vaginal hysterectomy.

**Table 4:** Relationship between major complications and the month of surgery.

Major complication	First 6 mo	6–12 mo	12–18 mo	Total
No injury	87	91	95	273
Bladder injury	0	2	0	2
Intestinal injury	1	0	0	1
Re-laparotomy	1	0	0	1
Total	89	93	95	277

*P* = .07.

**Table 5:** Relationship between minor complications and the month of surgery.

Minor complication	First 6 mo	6–12 mo	12–18 mo	Total
No complication	82	91	94	269
Wound infection	1	1	1	3
Hematuria	5*	0	0	5
Cuff hematoma	1	1	0	2
Total	89	93	95	277

*P* = .01.

* Most common minor complication.

## 4. Discussion

This study aimed to highlight the factors influencing surgical approach selection, stages of surgical development, and management of complications during the first 18 months of specialization in obstetrics and gynecology. Residency training in obstetrics and gynecology is a comprehensive process that involves multiple components. In gynecologic surgery, it is crucial to perform an adequate number of vaginal, abdominal, and laparoscopic hysterectomies to learn the appropriate techniques and develop skills in managing potential complications.^[5]^ Achieving these objectives requires training in high-volume centers under the guidance of experienced mentors. Moreover, integrating theoretical knowledge with practical applications and maintaining continuous effort are fundamental for acquiring and advancing surgical skills.

For many obstetricians and gynecologists, obstetrics constitutes the majority of their daily practices compared to gynecologic surgeries. At the beginning of specialization, particularly in centers with low volumes of gynecologic cases, gaining surgical experience can be challenging. Factors such as the surgeon’s future career aspirations, the quality of their training, and the conditions of the clinic in which they work significantly influence the number of cases they manage. The clinic discussed in this study, where the 2 surgeons gained experience during the first 18 months of their specialization, was a high-volume obstetrics and gynecology hospital with approximately 4000 annual deliveries and a busy obstetric workload.

The use of simulators plays a crucial role in obstetrics and gynecology training, particularly in laparoscopic surgery. Traditionally, animal and human cadaver laboratories have been considered highly valid due to their anatomical accuracy, tissue responsiveness, and haptic feedback. However, ethical concerns, regulatory restrictions, and high costs have led to a decline in their use.^[6]^ As an alternative, low-fidelity simulators, also known as box trainers, are widely utilized for developing laparoscopic surgical skills. These simulators offer significant advantages in improving instrument handling, enhancing hand-eye coordination, and adapting to progressively complex surgical scenarios.

Virtual reality-based simulators are increasingly integrated into surgical training, providing benefits such as time-motion analysis, real-time feedback, and objective performance evaluation. However, their widespread adoption is limited by high costs and technical infrastructure requirements.^[7]^ To enhance skill acquisition and improve surgical competency, the systematic integration of various simulation methods into laparoscopic surgery training is essential for optimizing the effectiveness of educational programs.

Approximately 500,000 hysterectomies are performed annually in the United States.^[8]^ Vaginal hysterectomy, as emphasized in numerous studies, is the preferred approach when feasible.^[9,10]^ During the initial 6 months, we avoided VH for non-prolapsed uteri and cases of total uterine prolapse. This was primarily due to the challenges in retraction during single-surgeon procedures and the higher level of surgical expertise required for non-prolapsed cases. In all prolapse cases, an abdominal approach incorporating mesh repair was favored. However, with increased surgical experience and confidence in the subsequent 6 months, vaginal hysterectomies were performed in non-prolapsed cases and even in patients with a history of cesarean delivery. Although the frequency of VH has declined with advancing technologies, it remains a safe and effective option for select patients.^[11]^ Due to the absence of a morcellator, TAH was preferred for cases with myomas larger than 10 cm. Despite the increased risk of intra-abdominal adhesions associated with a higher number of previous cesarean deliveries, laparoscopy is not the sole reason for conversion to laparotomy. We attribute this to the sufficient case volume that we managed to achieve during our residency training, which provided us with the necessary experience and confidence to handle such cases laparoscopically.^[12]^

The learning curve for TLH is reported to be approximately 75 cases in the literature, although the range may vary.^[13]^ Notably, the complication rates decreased significantly after this number. However, this technique is not free of complications. In our study, both surgeons had been practicing with a trainer box since their second year of residency. Consistent with the literature, we emphasize the importance of trainer boxes, particularly in enhancing suturing skills, in addition to case volume.^[14]^ We believe that the most critical factor in managing bladder and intestinal complications laparoscopically, rather than converting to laparotomy, is proficiency in suturing skills.

A study conducted in the United States reported that most residents completed their training, with experience in fewer than 18 vaginal hysterectomies.^[15]^ With the declining trend in VH procedures, it is imperative to ensure that this technique is taught during residency. Moreover, it should be remembered that, when feasible, VH remains the preferred method among hysterectomy techniques.

**Figure 1.**
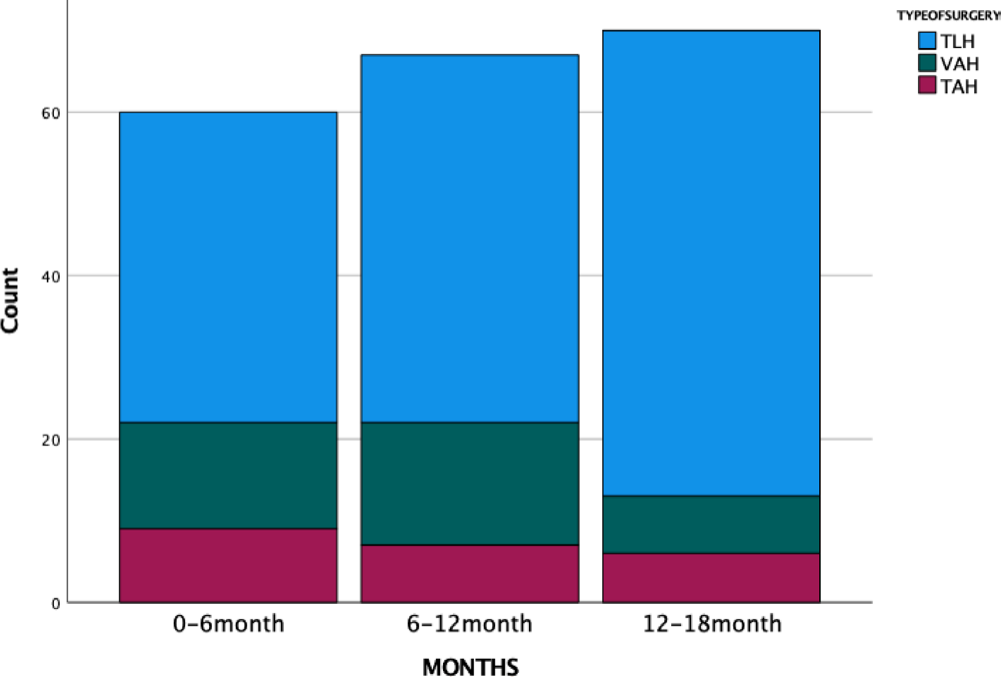
Distribution of hysterectomy types over the first 18 months of surgical practice.

When evaluating the first 18 months, we observed that TLH was the most frequently preferred method each 6-month period. Consistent with the literature, we believe that this preference is due to the advantages of TLH over laparotomy, including reduced pain, faster recovery, and better cosmetic outcomes.^[16]^ Over time, the proportional increase in TLH cases can be attributed to growing experience, enabling its use in more challenging cases such as large uteri, prior surgeries, and obesity (Fig. 1).

Major complications, including re-laparotomy (n = 1) and intestinal injury (n = 1), occurred in the TLH group during the first 6 months. In the 6 to 12 month period, bladder injuries were observed in 1 L/S Burch procedure and 1 VH case, both of which were successfully repaired laparoscopically. Despite having only 18 months of experience, we achieved complication rates consistent with the literature for major and minor complications in the TAH, VH, and TLH groups.^[17,18]^ In the literature, the rate of major complications in hysterectomy procedures has been reported to be approximately 4%. In our study, the incidence of major complications was determined to be 2% among a total of 197 hysterectomy cases. This lower complication rate may reflect the careful patient selection, meticulous surgical techniques, and the progressive improvement in perioperative management practices observed during the study period.^[19]^ This success appears to be associated with the number of cases performed during surgical training, regular practice with a trainer box, and extensive viewing of surgical videos.

In the first 18 months, we gained experience with V-NOTES, a technique recently gaining popularity in the literature for its advantages over conventional laparoscopic hysterectomy.^[20]^ Due to limited surgical equipment, we were able to perform only 4 cases, all without complications, which prevented us from making direct comparisons with other methods. In this technique, which combines vaginal and laparoscopic surgical skills, we observed that the assistant holding the camera plays a more active role than the assistant in laparoscopic hysterectomy, highlighting the need for a more experienced assistant in laparoscopy. For benign cases, the primary approach for hysterectomy should be the vaginal route whenever feasible. If this is not possible, laparoscopy should be the second preferred method. V-NOTES hysterectomy, however, should be positioned between these 2 techniques. It can be easily adopted by surgeons with sufficient experience in both vaginal and laparoscopic surgery. Regarding the learning curve, cumulative sum (CUSUM) analysis in the literature delineates 3 distinct phases: the competency phase (cases 1–5), the proficiency phase (cases 6–26), and the mastery phase (after the 31st case, involving the management of more complex cases).^[21]^ In our study, due to the limited number of V-NOTES cases, a direct comparison with other techniques could not be conducted. In early V-NOTES procedures, we observed that the most critical challenge in terms of procedural efficiency was identifying the anterior peritoneum and entering the abdominal cavity during anterior colpotomy, followed by the proper placement of the wound retractor.

In cases of total prolapse, we opted for combined laparoscopic total hysterectomy (TLH) and laparoscopic lateral suspension surgery. This technique has been described in the literature as a safe and effective alternative to sacrocolpopexy with low complication rates.^[22]^ The most significant limiting step in this procedure was the need for the assistant to play a more active role in securing the mesh, as well as the requirement for a large number of rapid laparoscopic sutures.

This study provides a comprehensive evaluation of the surgical performance of gynecology and obstetrics specialists during their first 18 months following their residency training. The collection of data under real-life conditions from a single center with a homogeneous patient group represents a significant advantage of the study. The variety of surgical procedures (such as TLH, VH, and abdominal hysterectomy) and comparisons between groups allow for an objective assessment of changes in surgical outcomes as experience increases. Additionally, the detailed examination of both major and minor complications offers valuable insights for improving clinical practices

The limitations of this study include the restricted number of patients in the study groups, which reduces the reliability of statistical analyses, particularly in smaller subgroups. Additionally, the low number of certain surgical procedures makes it difficult to draw meaningful conclusions about these techniques. Furthermore, the focus on data from only 2 surgeons may lead to the influence of individual performance differences on the results. While this study provides important findings based on real-world data regarding early surgical learning curves, its single-center design may limit broader applicability. Future multicenter analyses could offer a more comprehensive understanding of early surgical training processes across different healthcare settings.

This study provides a comprehensive analysis of the surgical experiences and learning processes of obstetrics and gynecology specialists during their first 18 months after completing residency training. The findings suggest that as surgical experience increases, complication rates decrease, and TLH gradually becomes the preferred approach. While complication rates were higher in the first 6 months, a significant decline was observed in the subsequent stages. However, despite being performed less frequently due to technological advancements, VH should be recognized for its importance and its role in surgical training should be preserved. This study offers valuable insights into the advancement of surgical expertise over time and the challenges encountered in the early stages of practice; however, these findings require validation through larger, multicenter studies..

## Author contributions

**Data curation:** Sercan Kantarci

**Formal analysis:** Sercan Kantarci, Alaattin Karabulut

**Investigation:** Sercan Kantarci

**Methodology:** Sercan Kantarci

**Supervision:** Sercan Kantarci

**Writing – original draft:** Sercan Kantarci

**Writing – review & editing:** Sercan Kantarci, Hüsnü Onur Durmaz
